# Who Are the Subjects with Gambling-Related Problems Requiring Treatment? A Study in Northern Italy

**DOI:** 10.3390/jcm7040080

**Published:** 2018-04-13

**Authors:** Raimondo Maria Pavarin, Angelo Fioritti, Silvia Marani, Daniele Gambini, Elsa Turino, Antonella Piazza

**Affiliations:** Epidemiological Monitoring Center on Addiction, Mental Health DSM-DP, Azienda USL, 40100 Bologna, Italy; angelo.fioritti@ausl.bologna.it (A.F.); s.marani@ausl.bologna.it (S.M.); d.gambini@ausl.bologna.it (D.G.); e.turino@ausl.bologna.it (E.T.); a.piazza@ausl.bologna.it (A.P.)

**Keywords:** pathological gambling, prevalence, mental health, alcohol, substance abuse

## Abstract

Background: This study analyzes data related to Hospital (HOS), Public Treatment Service Dedicated to Drug Addicts (SERD), or Community Mental Health Center (CMHC) clients with a first diagnosis of Pathological Gambling (PG) in the period 2000/2016 in Northern Italy. The aims were to describe trends and characteristics of pathological gamblers (PGs) and to estimate the prevalence of other diagnoses before or after the diagnosis of PG. Methods: Participants aged over 17 years with an ICD-9 or ICD-10 PG diagnosis were selected. Results: 680 PGs were identified, mean age 47.4 years, 20% female, 13% non-natives, 30% had other mental disorders diagnoses, 9% had alcohol dependence syndrome, and 11% had drug dependence. Most participants with comorbid disorders were diagnosed before PG, with a more elevated prevalence regarding mental disorders. Almost seven years had elapsed on average between the first admission and the diagnosis of PG. Conclusions: The results of this study highlight a growing demand for PG treatment addressed not only to SERD, but also to psychiatric and hospital services, based on the increase in SERD attendance from 2013. Many of them had already been treated for mental health problems before, but their percentage remained costant over time.

## 1. Introduction

Gambling disorders affect 0.2–5.3% of adults worldwide, although measurement and prevalence varies according to the screening instruments and methods used, and the availability and accessibility of gambling opportunities [1]; in Europe, it is between 0.12% and 3.4% [2,3,4,5]. In Italy the prevalence of subjects with gambling disorders among the general population (15/64 years) is between 1.3% [6] and 2.2% [7].

Over the last decade, most European countries have started a process of progressive legalization of gambling, which is still a national monopoly [8]. The Italian gambling market is the fourth largest in the world and the foremost in Europe, with total gross revenue of 96 billion Euros in 2016 [9].

A significant percentage of gamblers develops clinically relevant gambling problems [10], but only 10% seek treatment in clinic-based programs [11,12]. A large body of evidence indicates that pathological gambling (PG) is more likely among males, singles or divorcees, non-natives [13] people unemployed [14], with alcohol abuse [15], nicotine dependence [16], and is highly comorbid with other mental disorders [17,18] and substance [17] or alcohol dependence [1]. PG is indeed strongly associated with a range of psychiatric conditions, such as personality disorders, mood and anxiety disorders, and other impulse control disorders [19,20,21,22], suggesting that treatment for one condition should involve assessment and possible associated treatment for comorbid conditions [23]. Since pathological gamblers (PGs) experience high levels of mental comorbidity, screening for comorbid disorders upon entering treatment for gambling is recommended [24]. Likewise, routine PG screening in mental health services is strongly suggested, because PG seems to be over-represented and under-recognized in mental health populations [25,26]. 

In Italy, PGs can seek treatment in Services Dedicated to Drug Addicts (SERD), or in Community Mental Health Centers (CMHC) or in Hospital wards (HOS). In these three different public settings, treatment is covered by the National Health Service and is voluntary. In 2014 approximately 12,376 PGs attended a SERD [27] and this figure is estimated to have increased threefold since the beginning of SERD activities [28]. 

To improve treatment approaches, it is therefore necessary, besides a quantification/description of the phenomenon, to study the characteristics of persons seeking treatment for PG and to estimate the prevalence of other comorbid mental disorders, including alcohol and other substance use disorders.

The aims of this study, which analyzes data of the metropolitan area of Bologna (Northern Italy) related to HO, SERD, or CMHC users with a first diagnosis of PG in the period from 2000 to 2016, were (1) to describe trends and characteristics of PGs and (2) to estimate the prevalence of other diagnoses related to mental disorders and alcohol or substance dependence, comparing cases first diagnosed as PGs with those previously diagnosed with mental or alcohol/substance disorders.

## 2. Methods

Subjects aged over 17 years, referred for the first time to a HOS, a SERD, a CMHC with an ICD-9 (312.31) (ICD: International Classification of Diseases) or ICD-10 (F 63.0) diagnosis of PG, were selected. The population of the catchment area was about 860,000 inhabitants across the period of the study, from 2000–2016. We anonymously analyzed data from our routine activities. Personal identifiers were used following the rules of privacy regulation.

Data were retrospectively collected from the various digitalized archives: the PG diagnosis in HOS could have been primary or secondary, in SERDs it could be made at the first contact or in the subsequent periods, in CMHCs it could have been concomitant with other psychiatric diagnoses. The date of first access considered in this study refers to the first PG diagnosis.

Each person may have sought treatment in all these services and the information was collected at the first contact. The cases were selected from the IT systems of SERDs (9 units), CMHCs (11 units), and hospitals (10 facilities).

All the variables were drawn from the electronic databases: age, gender, country of birth, residence, diagnoses, services adressed, date of first contact, and of diagnostic assessments. 

To identify any other mental disorder, drug dependence, or alcohol dependence, the cohort was cross-checked with the electronic data available for all subjects referring, respectively, to the CMHCs, the HOS, and the SERDs in the metropolitan area of Bologna. According to the dates of the recorded diagnoses, the other disorders considered have been classified as occurring before or after the PG diagnosis.

### Statistical Analysis

All continuous and categorical variables were analyzed with a Student’s *t*-test and a chi-squared test, respectively.

To analyze the profiles of PGs who were first diagnosed for mental disorders, substance dependence and alcohol dependence, three multivariate analyses were performed using the logistic regression and the odds ratio was calculated along with the respective 95% confidence intervals [29].

Data analyses were performed using the STATA 15.0 statistical software program.

## 3. Results

### 3.1. Characteristics of PG

In the period from 2000 to 2016, 680 subjects attended a health facility for PG for the first time. Admissions had been rising steadily over time, but it should be noted that 68% occurred after 2012: five percent entered treatment in the period before 2005, 8% in the period 2005–2008, and 19% in the period 2009–2012.

As regards the first service accessed: 579 subjects (85%) were referred to a SERD, 77 (11%) to a CMHC, and 24 (4%) to a HOS. Over the whole period, some people (6%) turned to several services: 89% to a SERD, 13% to a CMHC, and 5% to a HO. 

Table 1 reports the characteristics of the incident cases in relation to the period of first admission. At first admission, mean age was 47.4 years, 20% were female, 13% non-natives, and 6% non-residents. Non-natives, mainly from Romania, Albania, Morocco, and Tunisia, were younger than Italians (mean age 39.2 vs. 48.6, *p* < 0.0001). The highest percentage of patients was in the 41–50 years age group. Females were older than men (mean age 54.5 vs. 45.6, *p* < 0.0001), with a greater percentage of cases over 50-years-old (67% vs. 33%, *p* < 0.0001). 

Socio-economic variables were available only for SERD and CMHC patients. As regards the educational level, it was reported that 10% had only completed primary school, 36% middle school, 31% had a high school diploma, and 4% were graduates. In relation to marital status, 34% were single, 33% married, 13% separated or divorced, 5% widows or widowers. As to professional status, 51% were employed, 14% unemployed, 16% were receiving a retirement or disability pension. In consequence of older age, widowhood (men 1% vs. women 18%) and retirement (men 12% vs. women 30%) were more frequent among women.

Overall, at least one subject in three was diagnosed with other mental disorders (depression, neurotic, and somatoform syndromes, and personality and behavioural disorders), more common among women (men 27% vs. women 47%) and natives (non-natives 10% vs. natives 34%).

Furthermore, 9% had alcohol dependence syndrome and 11% had drug dependence (4% cocaine, 3% heroin), which was more common among men (men 13% vs. women 3%).

After 2012 we highlight an increase in SERD clients, who represented 94% of all the new cases accessed. By comparison with the previous interval of time, we note a relevant increase in the 18–30 age class and a higher percentage of non-natives. There were no statistically significant differences regarding people with substance or alcohol dependence and with mental disorders, except for personality and behavioural disorders, who were decreasing.

### 3.2. Mental Disorders, Substance Dependence, and Alcohol Dependence before or after PG

Table 2 reports, for each problem, how many people were diagnosed before or after PG diagnosis.

Before PG, one in four were diagnosed with mental disorders, 8% with substance dependence and 7% with alcohol dependence. Among mental disorders, depressive disorders (12%), neurotic and somatoform syndromes (9%), personality and behavioural disorders (6%) were more frequent. The highest OR of having been diagnosed with a mental disorder before PG diagnosis was found for psychotic patients, apparently by reason of clinical severity.

On average, five years had passed from the first contact for substance dependence to the first diagnosis with PG, six years from the first contact for alcohol dependence, and almost seven years for mental disorders.

After first contact for PG, 6% of cases were diagnosed with mental disorders, 3% with substance dependence and 2% with alcohol dependence. For these subjects, on average, three years had passed from the first admission for PG to the subsequent contact for substance dependence, two years for alcohol dependence, and one year for mental disorders.

The odds of being diagnosed before PG with a mental disorder, a substance dependence, or a alcohol dependence were higher for all these problems, but more elevated for subjects with alcohol dependence syndrome. 

### 3.3. Multivariate Analysis

Three multivariate analyses were performed using a logistic regression to construct the profile of clients with mental disorders, substance dependence, and alcohol dependence diagnosed before PG. The variables used were sex, age group, nationality, period of first contact (2000–2012, 2013–2016), service of first contact for PG, alcohol dependence syndrome before PG, drug dependence before PG, and mental disorders before PG. The results are shown in Table 3.

In all the analyses, there appears to be no correlation with the period of the first admission. Otherwise, we noted a strong mutual relationship between mental disorders, alcohol dependence, and substance dependence.

Regarding the likelihood of having been first diagnosed with a mental disorder, the analysis revealed a positive association for PGs admitted to a hospital for the first time, women, natives, and subjects aged between 51 and 60 years. 

Men exhibited a higher likelihood of having been previously diagnosed with drug dependence, while a prior diagnosis of alcohol dependence was lower, but not statistically significant, for subjects who had their first contact before 2013.

## 4. Discussion

The findings of this study highlight a growing demand for PG treatment addressed not only to addiction units, but also to psychiatric and hospital services, based on the increase in SERD attendance from 2013. Many of these patients had already been treated for mental health problems before, but their percentage remained constant over time.

Most of the patients are males, aged from 40 to 50 years, 13% were born abroad, and at least 40% have a comorbid disorder: one in three suffers from mental disorders (especially depression), 11% from drug-dependence, and 9% from alcohol-dependence syndrome. 

As regards socio-economic status, while taking into account 22% of missing cases, we should point out the low level of education, the high presence of unemployed or retired subjects, and married or formerly married people. Sex, country of birth, and period of first contact are strongly associated with age and other patient characteristics. Men, especially non-natives, were younger and showed a higher frequency of drug-dependence. On the other hand, women were older, with a higher prevalence of retirees, widows, and other mental disorders. Furthermore, in the period after 2012, we highlight an important increase in SERD clients, non-natives, and subjects aged under 30. 

These figures are consistent with an array of international studies [30] and firmly support the need to improve cross-sector training and partnerships between mental health and addiction services. In fact, a growing body of evidence points out that PG is more likely to occur among males, single people, or divorcees, with alcohol abuse [15], non-natives [13], unemployed [14], and is highly comorbid with other mental disorders and drug or alcohol-dependence [31]. A substantial portion of gamblers in treatment is found to have co-occurring mental health disorders, including substance use disorders, especially alcohol-dependence, personality disorders, affective disorders, anxiety disorders, and impulse control disorders [32]. Among the PGs seeking treatment, men are more likely to use alcohol and illicit drugs, to be younger, white, and employed, as compared with females; females are instead more anxious and depressed [10].

Furthermore, other evidence highlights that treatment-seeking PGs may systematically differ from gamblers in the general population [24], insofar as they display more severe gambling-related symptoms and consequences, and a greater variety and intensity of co-morbid psychiatric disorders as compared with their non-treatment-seeking counterparts [12,18,20]. It should also be considered that a recent study shows that patients accessing mental health services are eight times more likely to be classified as PGs in relation to adult general population, that the mental health services should embed routine screening into clinical practice and train staff in the management of problem gambling [33].

For some authors, this may suggest a paradoxical effect, whereby problem gamblers with co-morbid disorders seek treatment at mental health or addiction services to manage their co-morbid psychopathology rather than at specialist gambling agencies for their gambling problems [24]. This implies that co-morbid psychiatric conditions may be of more concern than the gambling problem or that gamblers are more aware of their co-morbid symptomatology than their gambling symptomatology [20]. An alternative explanation is that the social acceptability, public awareness, and accessibility of mental health or addiction services may be higher than gambling services [20].

However, our study found that most subjects were diagnosed with comorbid disorders before being diagnosed with PG. This was true in particular for people with mental disorders, for whom almost seven years had elapsed on average between the first contact with the CMHC and the diagnosis of PG. Therefore, it is likely that this long delay in PG diagnosis confirms the tendency, reported by recent research, to under-recognize PG in mental health settings and indicates the need to reinforce referral pathways, screening abilities, and integrated treatment approaches across primary care, mental health, and addiction services [30]. In this regard, since brief assessment tools have been successfully developed for primary care and mental health settings, it should be both appropriate and straightforward to include such an effective screening in clinical interviews, in order to increase the opportunities for earlier PG identification and timely interventions.

Last but not least, the profile of clients with a diagnosis of mental disorders, substance-dependence, or alcohol-dependence before PG showed a strong mutual relationship between these previous disorders, with no correlation with the period of the first admission.

This study presents some limitations that reduce the generalizability of the results, hence further research is required involving specifically targeted studies. Since the data used are those available from the first admission, a great deal of information is lacking. The number of PGs might be underestimated in that, as our data are retrospective, there could be coding errors and the diagnosis might have been omitted or not have been transcribed in the digitized folder. Furthermore, it has not been possible to evaluate the data concerning different gambling habits, the use of tobacco, and of other substances, as they were not retrieved uniformly by the operators.

## 5. Conclusions

In conclusion, in Northern Italy, the number of people who were referred to services to treat PG has steadly increased, but the proportion of PGs with a previous diagnosis of mental disorders has remained high and stable over time. It follows that users who attend the services are not representative of the whole population of PGs, but only of a part, i.e., those more at risk and with other comorbid disorders. Moreover, the long delay in PG diagnosis for patients already treated for mental disorders confirms that PGs are still under-recognized in mental health settings.

This is a health and social problem that calls for further targeted research studies.

## Figures and Tables

**Table 1:** Pathological gambling: characteristics at the first admission.

	Total	%	2000–2012	%	2013–2016	%	*p*
Subjects	680	100.0	216	31.8	464	68.2	-
CMHC	77	11.3	60	27.8	17	3.7	<0.0001
SERD	579	85.2	141	65.3	438	94.4	-
HOS	24	3.5	15	6.9	9	1.9	-
Mean age	47.4	-	48.8	-	46.7		0.068
18–30 years	72	10.6	12	5.6	60	12.9	0.039
31–40 years	140	20.6	50	23.3	90	19.4	-
41–50 years	195	28.7	59	27.4	136	29.3	-
51–60 years	145	21.4	48	22.3	97	20.9	-
>60 years	127	18.7	46	21.4	81	17.5	-
Female	135	19.9	50	23.2	85	18.3	0.142
Non-natives	88	12.9	20	9.3	68	14.7	0.053
Substance dependence in the entire period	73	10.7	22	10.2	51	11.0	0.752
Cocaine	27	4.0	10	4.6	17	3.7	0.548
Heroin	17	2.5	6	2.8	11	2.4	0.752
Cannabis	5	0.7	2	0.9	3	0.7	0.691
Benzodiazepines	5	0.7	2	0.9	3	0.7	0.691
Alcohol dependence syndrome in the entire period	60	8.8	22	10.2	38	8.2	0.393
Mental disorders in the entire period	210	30.9	71	32.9	139	30.0	0.444
Schizophrenia and other functional psychoses	30	4.4	11	5.1	19	4.1	0.555
Mania and bipolar affective disorders	28	4.1	11	5.1	17	3.7	0.383
Depression	100	14.7	34	15.7	66	14.2	0.603
Neurotic and somatoform syndromes	72	10.6	25	11.6	47	10.1	0.569
Personality and behavioral disorders	58	8.5	28	13.0	30	6.5	0.005
Other psychic disorders	33	4.9	11	5.1	22	4.7	0.843

**Table 2:** Substance dependence, alcohol dependence, and mental disorders before/after pathological gambling.

	Subjects (680)				Before/After PG Univariate Analysis	
	Before PG	%	After PG	%	OR	95% CI
Substance dependence	54	7.9	19	2.8	2.84	1.68–4.79
Alcohol dependence syndrome	50	7.4	10	1.5	5.0	2.54–9.86
Mental disorders	167	24.6	43	6.3	3.88	2.78–5.43
Schizophrenia and other functional psychoses	29	4.3	1	0.2	29.0	3.95–212.89
Mania and bipolar affective disorders	24	3.5	4	0.6	6.0	2.08–17.29
Depression	83	12.2	17	2.5	4.88	2.90–8.23
Neurotic and somatoform syndromes	63	9.3	9	1.3	7.0	3.48–14.07
Personality and behavioral disorders	43	6.3	15	2.2	2.87	1.59–5.16
Other psychic disorders	29	4.3	4	0.6	7.25	2.55–20.62

**Table 3:** Mental disorders, drug dependence and alcohol dependence before pathological gambling: logistic regression analysis.

		Mental Disorders			Drug Dependence			Alcohol Dependence		
		OR	95% CI	*p*	OR	95% CI	*p*	OR	95% CI	*p*
Sex	Male	1	Referent	-	1	Referent	-	1	Referent	-
	Female	2.08	1.32–3.29	0.02	0.25	0.07–0.87	0.029	0.72	0.28–1.87	0.503
Age at first admission	18–30 years	1	Referent	-	1	Referent	-	1	Referent	-
	31–40 years	2.06	0.85–4.99	1.07	1.37	0.35–5.40	0.655	3.75	0.45–30.99	0.220
	41–50 years	1.76	0.76–4.11	1.89	2.84	0.80–10.03	0.105	6.62	0.86–51.01	0.069
	51–60 years	3.10	1.33–7.25	0.009	1.01	0.25–4.15	0.990	5.44	0.68–43.72	0.111
	>60 years	2.12	0.88–5.09	0.093	0.37	0.06–2.38	0.297	0.56	0.03–9.22	0.683
Nationality	Natives	1	Referent	-	1	Referent	-	1	Referent	-
	Non-natives	0.17	0.06–0.44	<0.0001	0.97	0.65–1.45	0.880	0.99	0.89–1.10	0.885
Period of first admission	2000–2012	1	Referent		1	Referent		1	Referent	
	2013–2016	1.39	0.88–2.18	0.154	1.20	0.56–2.56	0.642	0.51	0.26–1.02	0.059
First admission for PG	CMHC	1	Referent	-	1	Referent	-	1	Referent	-
	SERD	1.00	0.51–1.98	0.996	5.50	0.70–43.43	0.106	3.21	0.71–14.61	0.131
	HOS	7.64	2.55–22.93	<0.0001	2.99	0.23–38.13	0.400	4.14	0.59–29.04	0.153
Alcohol dependence syndrome before PG	No	1	Referent	-	1	Referent	-	1	Referent	-
	Yes	1.84	0.87–3.87	0.110	2.78	1.15–6.69	0.023	-	-	-
Drug dependence before PG	No	1	Referent	-	1	Referent	-	1	Referent	-
	Yes	3.69	1.90–7.18	<0.0001	-	-	-	2.76	1.15–6.61	0.023
Mental disorders before PG	No	1	Referent	-	1	Referent	-	1	Referent	-
	Yes	-	-	-	3.96	2.04–7.68	<0.0001	3.96	2.04–7.68	<0.0001
